# Covalent Organic Framework Enhanced Solid Polymer Electrolyte for Lithium Metal Batteries

**DOI:** 10.3390/molecules29081759

**Published:** 2024-04-12

**Authors:** Bingyi Ma, Lei Zhong, Sheng Huang, Min Xiao, Shuanjin Wang, Dongmei Han, Yuezhong Meng

**Affiliations:** 1 School of Chemical Engineering and Technology, Sun Yat-sen University, Guangzhou 510275, China; maby@mail2.sysu.edu.cn (B.M.); zhonglei8@mail.sysu.edu.cn (L.Z.); 2 The Key Laboratory of Low-Carbon Chemistry & Energy Conservation of Guangdong Province, State Key Laboratory of Optoelectronic Materials and Technologies, School of Materials Science and Engineering, Sun Yat-sen University, Guangzhou 510275, China; huangsh47@mail.sysu.edu.cn (S.H.); stsxm@mail.sysu.edu.cn (M.X.); wangshj@mail.sysu.edu.cn (S.W.); 3 Institute of Chemistry, Henan Academy of Sciences, Zhengzhou 450000, China; 4 Research Center of Green Catalysts, College of Chemistry, Zhengzhou University, Zhengzhou 450001, China

**Keywords:** solid polymer electrolyte, covalent organic frameworks, lithium metal batteries

## Abstract

High ionic conductivity, outstanding mechanical stability, and a wide electrochemical window are the keys to the application of solid-state lithium metal batteries (LMBs). Due to their regular channels for ion transport and tailored functional groups, covalent organic frameworks (COFs) have been applied to solid electrolytes to improve their performance. Herein, we report a flexible polyethylene oxide-COF-LZU1 (abbreviated as PEO-COF) electrolyte membrane with a high lithium ion transference number and satisfactory mechanical strength, allowing for dendrite-free and long-time cycling for LMBs. Benefiting from the interaction between bis(triflfluoromethanesulonyl)imide anions (TFSI^−^) and aldehyde groups in COF-LZU1, the Li^+^ transference number of the PEO-5% COF-LZU1 electrolyte reached up to 0.43, much higher than that of neat PEO electrolyte (0.18). Orderly channels are conducive to the homogenous Li-^+^ deposition, thereby inhibiting the lithium dendrites. The assembled LiFePO_4_|PEO-5% COF-LZU1/Li cells delivered a discharge specific capacity of 146 mAh g^−1^ and displayed a capacity retention of 80% after 200 cycles at 0.1 C (60 °C). The Li/Li symmetrical cells of the PEO-5% COF-LZU1 electrolyte presented a longer working stability at different current densities compared to that of the PEO electrolyte. Therefore, the enhanced comprehensive performance of the solid electrolyte shows potential application prospects for use in LMBs.

## 1. Introduction

With the growing demand in the electric vehicle market, people are committed to the development of lithium batteries with high safety, high energy, and high power density [1,2]. The commercial liquid electrolyte of lithium-ion batteries is mainly an organic solvent, which is flammable and leaky, bringing potential safety hazards [3,4]. In lithium metal batteries, an organic electrolyte can have adverse reactions with lithium [5]. During battery operation, an uneven Li^+^ flux distribution will lead to serious lithium dendrites, which will penetrate the separator and result in short circuiting and thermal runaway inside the batteries, thus causing a series of safety problems [6,7]. Solid-state batteries have recently attracted significant attention due to their high thermal stability and solid electrolyte with zero leakage [8]. Solid-state electrolytes are expected to solve the safety problems caused by liquid electrolytes [9,10]. Solid electrolytes include solid polymer electrolytes (SPEs) and solid inorganic electrolytes (SIEs) [11,12]. SPEs have attracted extensive attention due to their dimensional stability, process ability, low fabricating cost, and relatively small interface resistance [13,14]. Since Michel Armand proposed the first polymer electrolyte in the 1970s, polyethylene oxide (PEO) and its derivatives have been widely studied as the mainstream polymer electrolytes [15,16]. PEO presents the advantages of low cost, strong solvability, a high dielectric constant, and good compatibility with lithium anodes [17,18]. The ethylene oxide (EO) segment in PEO can interact with Li^+^ to promote the dissociation of lithium salts. It also conducts Li^+^ through long-term segmental mobility [19,20,21]. However, the chain mobility of crystalline PEO is weak, resulting in low ionic conductivity and a low lithium ion transference number at room temperature [22,23]. By modifying polymers through various methods such as grafting, blending, copolymerization, and crosslinking, SPEs with excellent physical, chemical, and electrochemical properties could be obtained [24].

Covalent organic frameworks are a class of porous materials connected by covalent bonds [25]. They have been widely used in catalysis, separation, sensing, energy storage, and so on [26]. The well-defined nanochannels of COFs are considered favorable transport channels. This means that COFs can provide a sufficient amount of free volume and number of stable channels for Li^+^ transport [27,28]. They guide the distribution of the lithium ion flux to regulate lithium deposition. Studies also have shown that nanosheets exfoliated from 2D COFs can provide abundant transport channels to enhance ion conductivity [29]. Moreover, the Li^+^ transference number and ionic conductivity can be increased by adjusting the functional groups of the COF [30]. Recently, researchers have been developed COF-based solid electrolytes with high performance [31,32,33]. For example, Wen-Yin Ko and coworkers designed a solid polymer electrolyte composed of PEO, LiClO_4_, and tetrakis (4-carboxyphenyl) porphyrin (TCPP), a kind of porphyrin COF [34]. TCPP can effectively inhibit the crystallinity of PEO and thus improve its ionic conductivity. Zhang et al. constructed COF-poly(vinyl ethylene carbonate) (PVEC) (COF-PVEC) electrolyte membranes by grafting PVEC onto COF-V channels [35]. The triazine group of COF-V can enhance the Li^+^ transport. As cross-linking sites, COF-V form a network framework to improve mechanical properties. Lu et al. reported a vinylene-linked covalent organic framework (VCOF)-based SPE composed of VCOF-1, PEO, PVDF, and LiTFSI [1]. The cylindrical channel of VCOF-1 was conducive to the migration of lithium ions. When <4 wt% VCOF-1 was added, the ion conductivity increased by 25% and the transference number of lithium ions increased by 46%. Moreover, the VCOF-SPE exhibited superior mechanical properties, with a tensile strength of 1.43 MPa, and was beneficial for suppressing the growth of lithium dendrites. Atsushi Nagai et al. focused on the application of COF materials in solid-state batteries and studied the impact of different types of bonds on the key performance of batteries [36]. Building blocks and two-dimensional or three-dimensional pore structures of COFs are crucial for improving the ion conductivity or charge mobility. The structural stiffness of COFs should be moderate. A structure that is too rigid can hinder ion migration, while being too flexible can damage the mechanical integrity and affect the operation of batteries. In addition, active centers within COFs can promote the dissolution of lithium salts, thereby promoting ion conduction.

The above studies indicate that blending polymers and COFs can improve the electrochemical performance of SPEs. However, the severe agglomeration of fillers results in local inhomogeneity and consequently a tortuous path for Li ion diffusion with a decreased ionic conductivity [37]. Herein, we report a flexible solid polymer electrolyte membrane composed of PEO, bistrifluoromethanesulfonimide lithium salt (LiTFSI), and COF-LZU1, which was prepared using the doctor blade technique. Instead of directly blending COFs with PEO, the monomers used to synthesize COF-LZU1 were added to the polymer solution and scraped onto a PTFE pad to generate COF-LZU1 in situ, ensuring the uniform generation of COF-LZU1 in the electrolyte. COF-LZU1 is linked by imine bonds and contains aldehyde groups [38]. Its ordered channels facilitate the diffusion of lithium ions and regulate lithium deposition, so it can inhibit lithium dendrite growth and improve the anode interface compatibility. Moreover, the aldehyde groups interact with TFSI^−^ and restrict the movement of TFSI^−^, hence increasing the Li^+^ transference number [39]. The electrochemical performance is effectively improved. Compared to the PEO electrolyte, the PEO-5% COF-LZU1 electrolyte presented an ionic conductivity of 3.30 × 10^−4^ S cm^−1^ and a higher Li^+^ transference number of 0.43 (60 °C). The LiFePO_4_/Li cell delivered an initial discharge specific capacity of 146 mAh g^−1^ with a capacity retention of 80% after 200 cycles at 0.1 C (60 °C). The Li/Li symmetrical cell of the PEO-5% COF electrolyte worked stably for more than 350 h with a low overvoltage at 0.1 mA cm^−2^ (60 °C). Therefore, the designed PEO-COF solid electrolyte provides a reference for the study of solid electrolytes for LMBs.

## 2. Results and Discussion

### 2.1. Physico-Chemical Characterization

**Figure 1:**
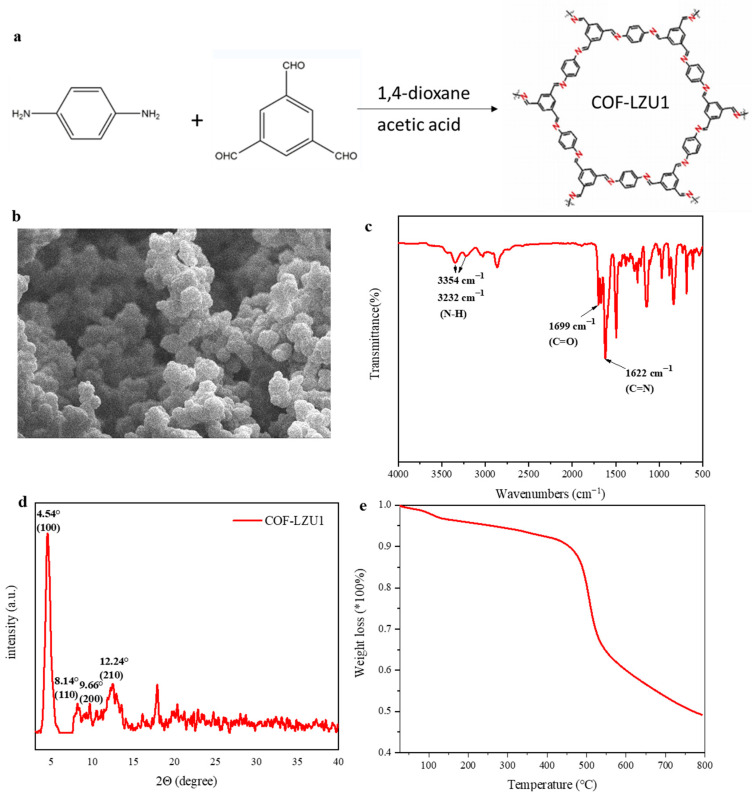
(**a**) Synthesis of COF-LZU1. (**b**) SEM image, (**c**) FTIR spectrum, (**d**) XRD pattern, and (**e**) thermogravimetric analysis of COF-LZU1.

COF-LZU1 was obtained via a Schiff base reaction with 1,3,5-triformylbenzene and 1,4-diaminobenzene (Figure 1a). As shown in Figure 1b, the morphology of COF-LZU1 showed agglomeration particles, with a particle size of about 1 μm. To demonstrate the successful synthesis of COF-LZU1, Fourier-transform infrared spectroscopy and X-ray diffraction were conducted. As shown in Figure 1c, the C=N stretching band from imine at 1622 cm^−1^ appeared, indicating the successful synthesis of COF-LZU1. The peaks at 1699 cm^−1^ and 3354 cm^−1^ represent the N-H stretching band of amino groups and the C=O bond of aldehyde groups, respectively. It was obvious that monomers did not require or could not undergo a complete reaction, and only COF-LZU1 was generated. The remaining monomers contained aldehyde groups, which can interact with lithium anions and increase the migration number of lithium ions. Moreover, the X-ray patterns of COF-LZU1 showed a strong diffraction peak at 4.54°, with a few weak peaks at 8.14°, 9.66°, and 12.24°, assigned to (100), (110), (200), and (210) diffractions (Figure 1d). The thermogravimetric result of COF-LZU1 reveals a thermal stability of about 400 °C (Figure 1e).

**Figure 2:**
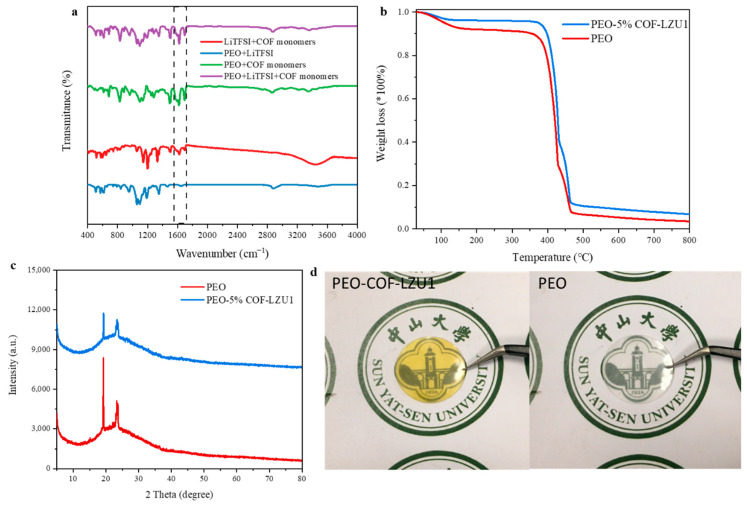
(**a**) FTIR spectrum of electrolyte membrane composed of different materials. (**b**) Thermogravimetric analysis results, (**c**) XRD test results, and (**d**) images of PEO-5% COF-LZU1 electrolyte and PEO electrolyte.

The PEO-COF electrolyte membrane was prepared by the doctor blade technique. Instead of blending the polymer and COF-LZU1 powder directly, the monomers were added to the polymer solution and then scraped onto a Teflon plate. The COF-LZU1 was synthesized in situ in the scraped film. This not only avoided the problem of uneven mixing of the COF powder, but also simplified the operation. To confirm the formation of COF-LZU1 in the electrolyte membrane, Fourier-transform infrared spectroscopy was performed under different conditions. As shown in Figure 2a, when the monomers were added, a characteristic peak appeared at 1620 cm^−1^, corresponding to C=N stretching of COF-LZU1 (purple, green, and red curves), while no C=N stretching band appeared when the monomer was not added (blue curve). As shown in Figure 2d, the PEO-COF membrane (on the left) was transparent and yellow and the PEO membrane (on the right) had a thickness of approximately 100 μm. Thermogravimetric curves (Figure 2b) showed that the thermal decomposition temperature of the PEO-COF electrolyte was slightly higher than that of the PEO membrane, which may be attributed to the heat resistance of COF-LZU1. To eliminated the possibility of the material in the thin film being an amorphous polymer, XRD tests were conducted on the PEO electrolyte and PEO-5% COF-LZU1 electrolyte (Figure 2c). It can be seen the peak intensity of PEO-5% COF-LZU1 decreased compared to that of the PEO electrolyte, indicating a decrease in crystallinity, which is beneficial for ion migration. Overall, it was still an amorphous polymer.

### 2.2. Electrochemical Characterization

**Figure 3:**
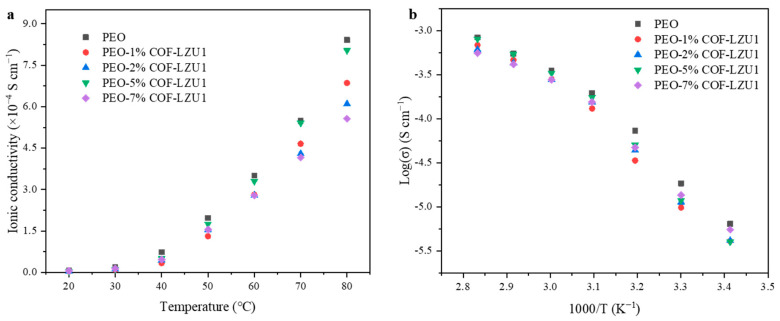
(**a**) Variation in conductivity with temperature and (**b**) VTF plots of the ionic conductivities for the prepared films with different concentrations of COF-LZU1 monomer.

To explore the ionic conductivity, solid polymer electrolytes with different COF monomer contents were tested at 30–80 °C. According to the mass content of the COF-LZU1 monomer, the obtained SPEs were named PEO, PEO-1% COF-LZU1, PEO-3% COF-LZU1, PEO-5% COF-LZU1, and PEO-7% COF-LZU1. As shown in Figure 3a and Table 1, the ionic conductivities of the PEO, PEO-1% COF-LZU1, PEO-3% COF-LZU1, PEO-5% COF-LZU1 and PEO-7% COF-LZU1 electrolytes were 3.50 × 10^−4^, 2.81 × 10^−4^, 2.79 × 10^−4^, 3.30 × 10^−4^, 2.78 × 10^−4^ at 60 °C, respectively. After introducing COF-LZU1, the ionic conductivity decreased slightly, and ion transport was hindered when the content of the COF monomer reached 7% compared to 5% COF-LZU1. This phenomenon could be explained by the “permeation effect”. It can be seen from Figure 3b that all the electrolytes showed Vogel–Tamman–Fulcher (VTF)-like plots of ionic conductivity (σ) versus T^−l^, indicating the same ion transport mechanism.

**Figure 4:**
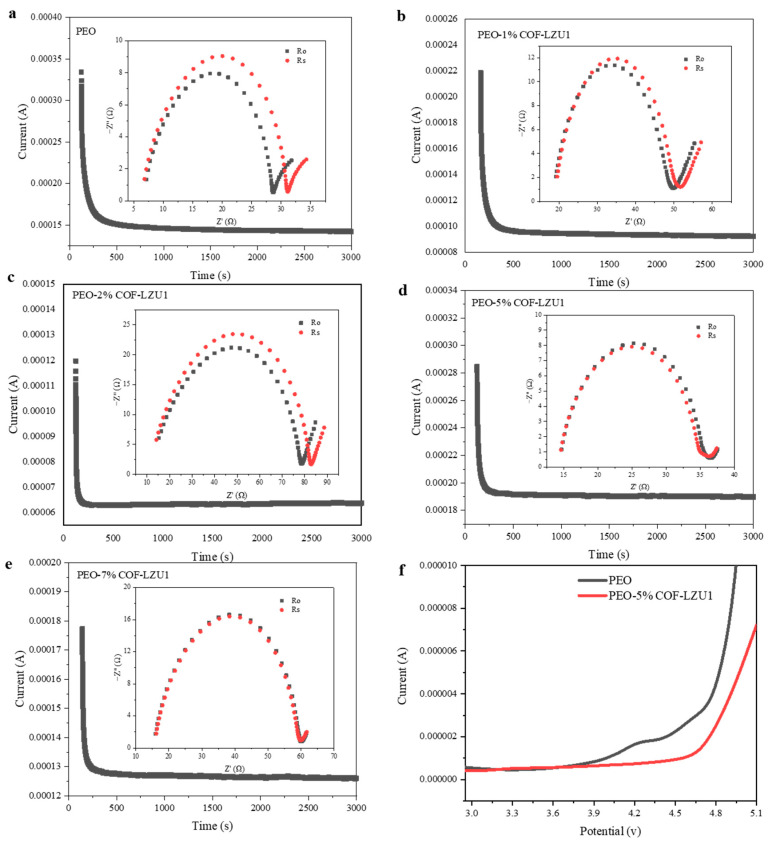
Chronoamperometry profile of (**a**) Li/PEO/Li, (**b**) Li/PEO-1% COF-LZU1/Li, (**c**) Li/PEO-2% COF-LZU1/Li, (**d**) Li/PEO-5% COF-LZU1/Li, and (**e**) Li/PEO-7% COF-LZU1/Li symmetric cells under a polarization voltage of 10 mV, and the EIS before and after the polarization (inset). (**f**) LSV profile of PEO-5%COF-LZU1 and PEO electrolytes.

The lithium ion transference number is an important index to evaluate the electrochemical performance of SPEs. It can be measured using AC impedance spectra and the steady-state current technique. As shown in Figure 4a–e, the Li^+^ transference numbers of PEO, PEO-1% COF-LZU1, PEO-2% COF-LZU1, PEO-5% COF-LZU1, and PEO-7% COF-LZU1 were 0.18, 0.18, 0.19, 0.43, and 0.35 at 60 °C, respectively. The pure PEO electrolyte had a strong chelating ability for lithium ions by oxygen atoms, resulting in a strong binding effect of lithium ions and a low Li^+^ transference number. After introducing COF-LZU1, the Li^+^ transference number significantly increased, for two main reasons. Firstly, COF-LZU1 can promote the relaxation of the polymer molecular chains and reduce their crystallinity, inhibiting the movement of anions. Secondly, the aldehyde group can interact with lithium anions through Lewis acid–base interactions, effectively anchoring TFSI^−^ and inhibiting its movement. At the same time, it promotes the dissociation of lithium salts, increase the number of freely moving lithium ions in the system, and thus improve the Li^+^ transference number. Based on its ion conductivity and lithium ion transference number, the PEO-5% COF-LZU1 electrolyte was selected for further study. The electrochemical stability window of PEO-5% COF-LZU1 was evaluated by linear sweep voltammetry at 60 °C (Figure 4f). The electrochemical decomposition potential of PEO-5% COF-LZU1 was 4.6 V, slightly higher than that of PEO (3.8 V), which may due to the introduction of COF-LZU1 to stabilize the PEO chain segment and preventing the decomposition of the polymer at high voltages. COF-LZU1 helped to improve the compatibility between solid electrolytes and high-voltage cathode materials, and thus has broad application prospects in high-voltage solid-state lithium batteries.

**Figure 5:**
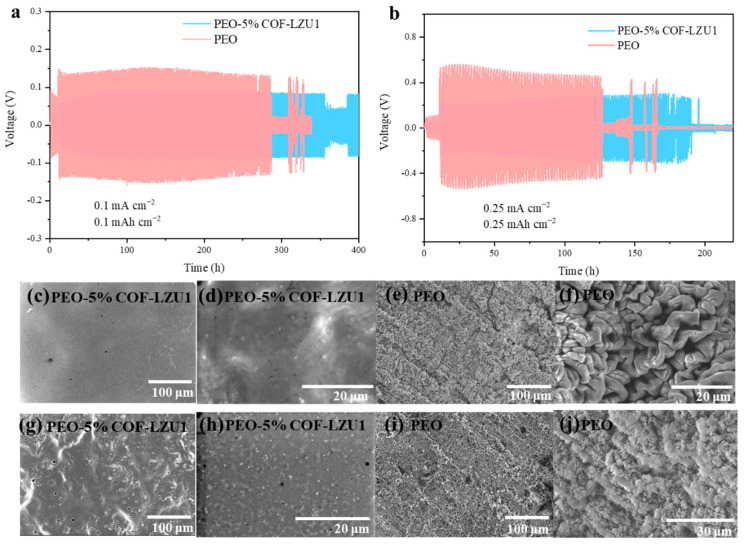
Electrochemical performance of Li/PEO-5%COF-LZU1/Li symmetric cell and Li/PEO/Li symmetric cell with a current density of (**a**) 0.1 mA cm^−2^ and (**b**) 0.25 mA cm^−2^ with a capacity of 1 mAh cm^−2^. SEM images of cycled lithium metal anode collected from the Li/PEO-5% COF-LZU1/Li symmetric cell with a current density of (**c**,**d**) 0.1 mA cm^−2^ for 100 h and (**g**,**h**) 0.25 mA cm^−2^ for 80 h, and Li/PEO/Li symmetric cell at (**e**,**f**) 0.1 mA cm^−2^ for 100 h and (**i**,**j**) 0.25 mA cm^−2^ for 80 h.

To verify their cycling stability and ability to inhibit lithium dendrites, Li-Li symmetric batteries with the PEO-5% COF-LZU1 electrolyte and PEO electrolyte were assembled and tested at 60 °C. At a current density of 0.1 mA cm^−2^, the Li/PEO/Li battery exhibited a high polarization voltage (130 mV), with a significant decrease in polarization voltage at around 280 h, indicating that the uneven accumulation of lithium dendrites caused battery short circuits (Figure 5a). On the other hand, the Li/PEO-5% COF-LZU1/Li battery exhibited stable cycling for more than 350 h and maintained a lower stable polarization potential (85 mV). This indicates the formation of a stable interface between the lithium metal and PEO-5% COF-LZU1 electrolyte, which exhibited a certain mechanical strength and could suppress the growing of lithium dendrites. We disassembled the lithium symmetric battery after cycling for a period of time and observed the morphology of the lithium metal electrode. It can be seen from the SEM images that after cycling for 100 h, the surface of the lithium anode of the Li/PEO-5% COF-LZU1/Li battery was smooth (Figure 5c,d), while the surface of the lithium anode of the Li/PEO/Li battery had more cracks and dendrites (Figure 5e,f). When the current density was further increased to 0.25 mA cm^−2^, the Li/PEO-5% COF-LZU1/Li battery presented a lifespan of 190 h and a voltage hysteresis of 0.3 V, while the Li/PEO/Li battery presented a shorter lifespan of 127 h and a higher voltage hysteresis of 0.5 V (Figure 5b). After 80 cycles, the metallic Li of the PEO electrolyte became loose and many lithium dendrites formed, while the lithium of the PEO-5% COF-LZU1 electrolyte remained smooth (Figure 5g–j). The above results indicated that COF-LZU1 could effectively constrain the formation of lithium dendrites, mainly because the regular ionic channel of COF-LZU1 regulated the deposition of lithium, resulting in a stable interphase. However, as the current density increased, the interface stability between the solid electrolyte and lithium anode decreased, indicating that the PEO-5% COF-LZU1 electrolyte can only improve the interface stability at low current densities.

**Figure 6:**
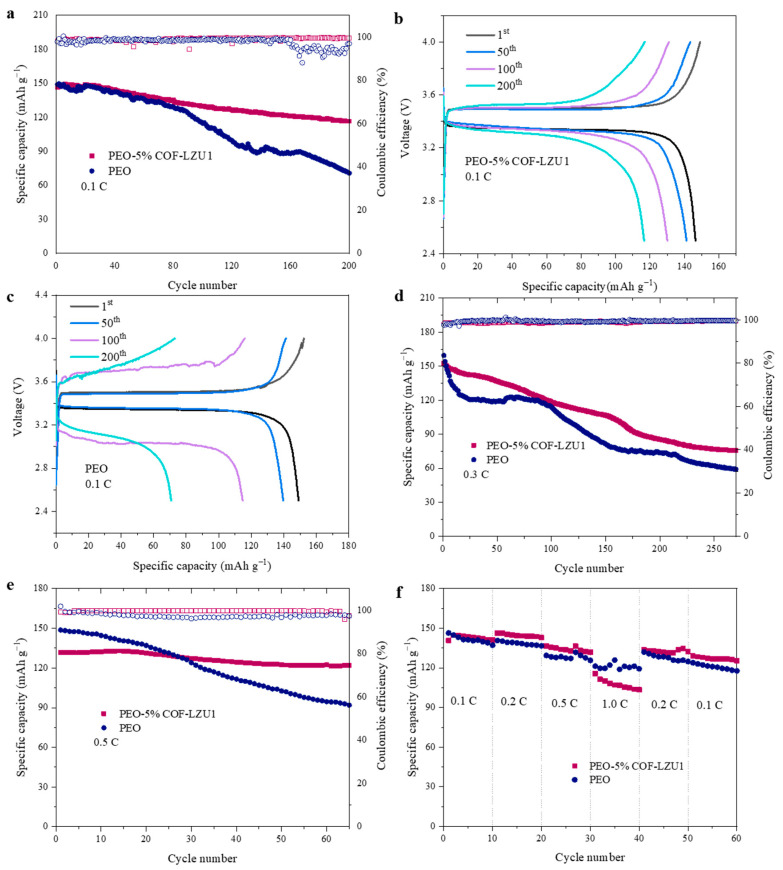
Long cycling performance of LFP/PEO-5% COF-LZU1/Li and LFP/PEO/Li cells at (**a**) 0.1 C, (**d**) 0.3 C, and (**e**) 0.5 C at 60 °C. Galvanostatic charge/discharge voltage profiles at different cycles for (**b**) LFP/PEO-5% COF-LZU1/Li cell and (**c**) LFP/PEO/Li cell at 0.1 C. (**f**) Rate performance of LFP/PEO-5% COF-LZU1/Li and LFP/PEO/Li cells at 60 °C.

To further explore the long cycling properties and rate performance, 2025-type coin cells of LiFePO_4_ (LFP)/Li were assembled and tested at 60 °C. Figure 6a shows the cycle performance of cells with the PEO-5% COF-LZU1 and PEO electrolytes at 0.1 C with a cathode LFP content of about 1.5 mg cm^−2^. LFP/PEO-5% COF-LZU1/Li delivered a discharge specific capacity of 146 mAh g^−1^ with a capacity retention of 80% after 200 cycles, while the capacity retention of LFP/PEO/Li was 48% after 100 cycles. PEO-5% COF-LZU1 presented a stable cycling with an overpotential of 0.2 V (Figure 6b), suggesting a small polarization. However, the polarization of LFP/PEO/Li increased with the number of cycles, resulting in unstable batteries (Figure 6c). During the first cycle of charging, there was an observed spike in the peak of LFP/PEO/Li, which may be due to the unstable charging and discharging process in the first cycle of the battery and the formation of the SEI. After polarization completed and a stable SEI formed, the battery can stably charge and discharge. Tested at 0.3 C with an LFP content of about 3.5 mg cm^−2^ after 200 cycles, the initial discharge capacity and the capacity retention of PEO-5% COF were 152 mAh g^−1^ and 56%, while those of the PEO batteries were 159 mAh g^−1^ and 46% (Figure 6d). When tested at 0.5 C with an LFP content of 3.5 mg cm^−2^, the capacity retention of the LFP/PEO-5% COF-LZU1/Li cell and LFP/PEO/Li cell were 92% and 64% after 60 cycles, respectively, with an average coulombic efficiency of 100% and 97% (Figure 6e). Figure 6f shows the rate properties of LFP/PEO-5% COF-LZU1/Li and LFP/PEO/Li cells at 0.1 C, 0.2 C, 0.5 C, 1 C, 0.2 C, and 0.1 C. When the current density returned to 0.1 C, compared with that at the initial 0.1 C, the capacity attenuation was relatively low for the LFP/PEO-5% COF-LZU1/Li cell. However, at the high rate of 1C, the specific capacity of the LFP/PEO-5% COF-LZU1/Li cell was slightly lower than that of the LFP/PEO/Li cells, and the coulombic efficiency decreased rapidly. This means that COF-LZU1 produced a limited improvement in rate performance. Notably, at 0.5 C, the specific capacity increased in both cases, and at 0.2 C, this was only observed in PEO-5% and not in PEO. This could be related to the destruction and recombination of SEI at different rates. When the current density increased from 0.1 C to 0.5 C, the SEI may be damaged and then it forms a stable SEI again. Therefore, a small increase in specific capacity was observed at 0.5 C. At 0.2 C, the PEO-5% COF-LZU1 battery also experienced an increase in the specific capacity for the same reason, while the SEI of the PEO battery was not damaged.

## 3. Materials and Methods

### 3.1. Materials

1,3,5-triformylbenzene was purchased from BIDE (Jiangyin, China). 1,4-diaminobenzene, bistrifluoromethanesulfonimide lithium salts, and anhydrous acetonitrile were obtained from Macklin (Shanghai, China). 1,4-dioxane was purchased from Meryer (Shanghai, China). PEO was purchased from Sigma Aldrich (Shanghai, China). Dimethylformamide was obtained from acros (Guangzhou, China). Acetic acid was purchased from Aladdin (Shanghai, China).

### 3.2. Synthesis of COF-LZU1

According to previous reports [38,39], one monomer (1,3,5-triformylbenzene; 0.75 g) was dissolved in 40 mL 1,4-dioxane and then the other monomer (1,4-diaminobenzene; 0.75 g) was added. After 20 min of ultrasound treatment and uniform dispersion, a 3.0 mol/L acetic acid aqueous solution (8 mL) was slowly added. It was then left to react for 3 days at room temperature. The precipitate was collected by centrifugation, washed with dimethylformamide 5 times, and vacuum dried overnight at 60 °C to obtain the final product (COF-LZU1).

### 3.3. Preparation of Solid Polymer Electrolyte Membrane

First, PEO (Mw = 600,000) and LiTFSI were dissolved in anhydrous acetonitrile with an O/Li molar ratio of 10:1, and the mixture was named solution A. The monomer 1,3,5-triformylbenzene and 1,4-diaminobenzene were each dissolved in a small amount of anhydrous acetonitrile. The 1,3,5-triformylbenzene solution was dropped into solution A and after magnetic stirring, the 1,4-diaminobenzene solution was added to the mixture and stirred for 5 min. The mixture was quickly scraped onto a Teflon pad through the doctor blade technique. It was left to react for two days, and then the solvent was slowly evaporated at room temperature and then completely removed by incubation at 60 °C. A membrane with a thickness of 100 μm was finally peeled off from the Teflon pad. The mass contents of the added COF monomer were 0%, 1%, 3%, 5%, and 7%, respectively. All the above steps were conducted in a glove box in an argon atmosphere. The content of O_2_ and H_2_O was less than 0.1 ppm.

### 3.4. Preparation and Modification of LFP Electrodes

An appropriate amount of LFP (70 wt%), Super P (1 wt%), PEO (M_w_ = 100,000, 1.4 wt%), and LiTFSI (0.6 wt%) were added to a DMF/ACN mixed solvent. Then, the mixture was placed in a planetary mill and ball milled at 150 rpm for 12 h to obtain a uniformly flowing slurry. A scraper was used to scrape the slurry onto the surface of carbon-coated aluminum foil. Subsequently, the coated aluminum foil was transferred to a 50 °C blast oven to dry for 1 h, and then it was transferred to a 60 °C vacuum oven to dry for 24 h to ensure complete solvent removal. The dried aluminum foil was cut into circular electrode plates with a diameter of 12 mm using a laminating machine (-Kejing, Shenzhen, China).

To ensure the interface compatibility between the electrode and electrolyte, surface modification of the LFP electrode was required before assembling the battery. The specific method was to dissolve the PEO/LiTFSI in a certain proportion of a DMF/ACN solvent; 15 microliters of the mixture was then used to evenly coat the surface of the electrode. It was left to stand overnight to evaporate the solvent.

### 3.5. Characterizations

The morphology and surface of the materials were analyzed using a scanning electron microscope (SEM, HITACHI S4800, Hitachi Ltd., Tokyo, Japan). The crystal structure of COF-LZU1 was studied using an X-ray diffractometer (XRD, Dmax 2200, Rigaku Ltd., Tokyo, Japan) with Cu Kα radiation (λ = 0.15418 nm). The functional groups of the samples were confirmed by Fourier-transform infrared spectroscopy (FTIR, Analect Company, New York, NY, USA). Thermogravimetric analyses (TGAs) were performed using a PerkinElmer Pyris Diamond TG/DTA analyzer (PerkinElmer, Waltham, MA, USA) at a heating rate of 10 °C min^−1^ in the temperature range of 30–800 °C.

### 3.6. Cell Assembly and Electrochemical Characterization

Coin cells were assembled to perform electrochemical measurements. The assembly of the cells was carried out in a glove box filled with an argon atmosphere. The content of O_2_ and H_2_O was less than 0.1 ppm. CR2032 or CR2025 solid-state coin cells were assembled according to testing requirements: LFP/solid electrolyte/Li, Li/solid electrolyte/Li, steel sheet/solid electrolyte/steel sheet, and carbon paper electrode/solid electrolyte/Li. As an example, the assembly of the LFP/solid electrolyte/Li cells were assembled in the order of “positive electrode shell—LFP electrode—electrolyte film—lithium anode—steel sheet—shim—negative electrode shell”, followed by a sealing treatment. After assembly, the cells were placed in a 60 °C blast oven for 24 h to enhance the interface stability between the electrode and electrolyte.

The cells were tested using a Wuhan LAND battery system. The ionic conductivity was measured by assembling stainless-steel (SS)/electrolyte membrane/SS for AC impedance spectroscopy measurements in the frequency range of 1 Hz–0.1 M Hz. The lithium transference number was measured by assembling Li/electrolyte membrane/Li through the AC impedance with DC polarization method, with ΔV = 10 mV. Linear sweep voltammetry was performed by assembling carbon paper electrode/electrolyte membrane/Li at a scan rate of 1 mV S^−1^ from the open circuit voltage to 6 V. The ionic conductivity, lithium transference number, and current were tested using an electrochemical workstation (Metrohm, Herisau, Switzerland).

## 4. Conclusions

In this work, we developed a flexible PEO-COF-LZU1 electrolyte membrane to enhance the electrochemical performance of solid-state lithium metal batteries. A PEO-COF electrolyte was generated in situ on a polytetrafluoro pad using a scraping method, which not only avoided the uneven dispersion of COF-LZU1 particles but also simplified the preparation process. Meanwhile, the generation of COF particles also enhanced the mechanical stability of the electrolyte membrane to a certain extent. Thanks to the fast ion channels provided by COF-LZU1, the ionic conductivity of the PEO-5% COF-LZU1 membrane was 3.30 × 10^−4^ S cm^−1^ at 60 °C. The significant increase in Li^+^ transference number of 0.43 could be attributed to the interaction between the aldehyde group and TFSI^−^, thus immobilizing the anion. Furthermore, the channel of COF-LZU1 helped to regulate the deposition of lithium and effectively inhibited lithium dendrite growth. Therefore, the Li/Li symmetric batteries delivered a stable cycle of more than 350 h with a voltage hysteresis of about 85 mV at 0.1 mA cm^−2^. The full battery performance was also comprehensively improved. The discharge capacity retention rates of the LFP/PEO-5% COF-LZU1/Li cell were 80% and 92% at 0.1 C and 0.5 C after 200 cycles and 60 cycles, respectively, higher than those of LFP/PEO/Li (48%, 64%). Our research provides a strategy for developing PEO-COF electrolytes with good performance for solid-state lithium metal batteries.

## Figures and Tables

**Table 1:** Ionic conductivity of different electrolyte membranes at 30–80 °C.

	30 °C (S cm^−1^)	40 °C (S cm^−1^)	50 °C (S cm^−1^)	60 °C (S cm^−1^)	70 °C (S cm^−1^)	80 °C (S cm^−1^)
PEO	1.85 × 10^−5^	7.73 × 10^−5^	1.97 × 10^−4^	3.50 × 10^−4^	5.49 × 10^−4^	8.41 × 10^−4^
PEO-1% COF-LZU1	9.84 × 10^−6^	3.36 × 10^−5^	1.31 × 10^−4^	2.81 × 10^−4^	4.66 × 10^−4^	6.86 × 10^−4^
PEO-2% COF-LZU1	1.14 × 10^−5^	4.39 × 10^−5^	1.54 × 10^−4^	2.79 × 10^−4^	4.30 × 10^−4^	6.10 × 10^−4^
PEO-5% COF-LZU1	1.89 × 10^−5^	5.06 × 10^−5^	1.75 × 10^−4^	3.30 × 10^−4^	5.41 × 10^−4^	8.04 × 10^−4^
PEO-7% COF-LZU1	1.36 × 10^−5^	4.74 × 10^−5^	1.55 × 10^−4^	2.78 × 10^−4^	4.16 × 10^−4^	5.56 × 10^−4^

## Data Availability

The raw data supporting the conclusions of this article will be made available by the authors on request.
